# Supplementary figures and images for: The Fungal Effector Mlp37347 Alters Plasmodesmata Fluxes and Enhances Susceptibility to Pathogen
Source: Microorganisms. 2021 Jun 6;9(6):1232. doi: 10.3390/microorganisms9061232 (PMC8228402; doi:10.3390/microorganisms9061232)

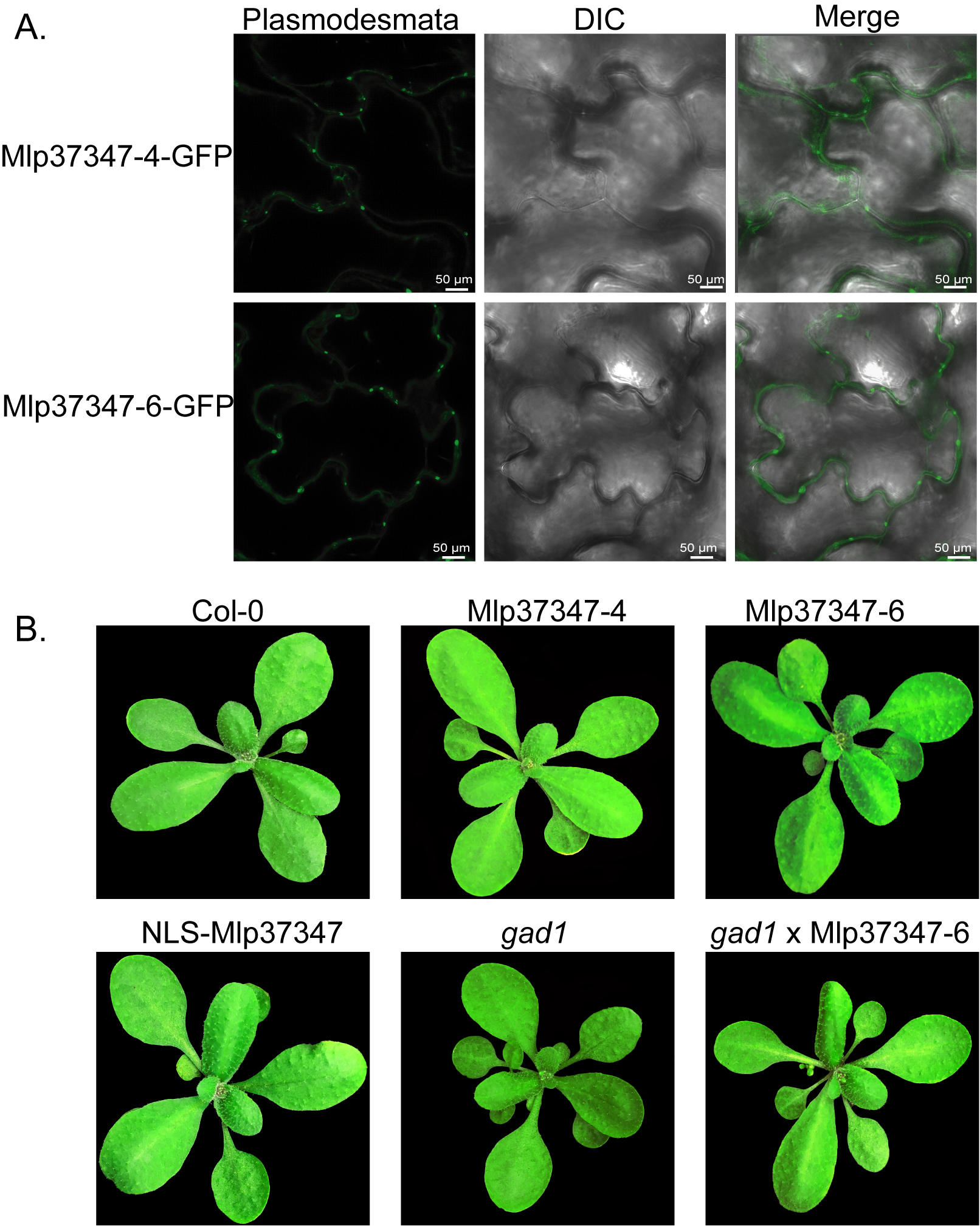

Supplement: Supplementary file 1 [file microorganisms-09-01232-s001.zip › microorganisms-1239006-supplementary/Supp Fig 1.tif]

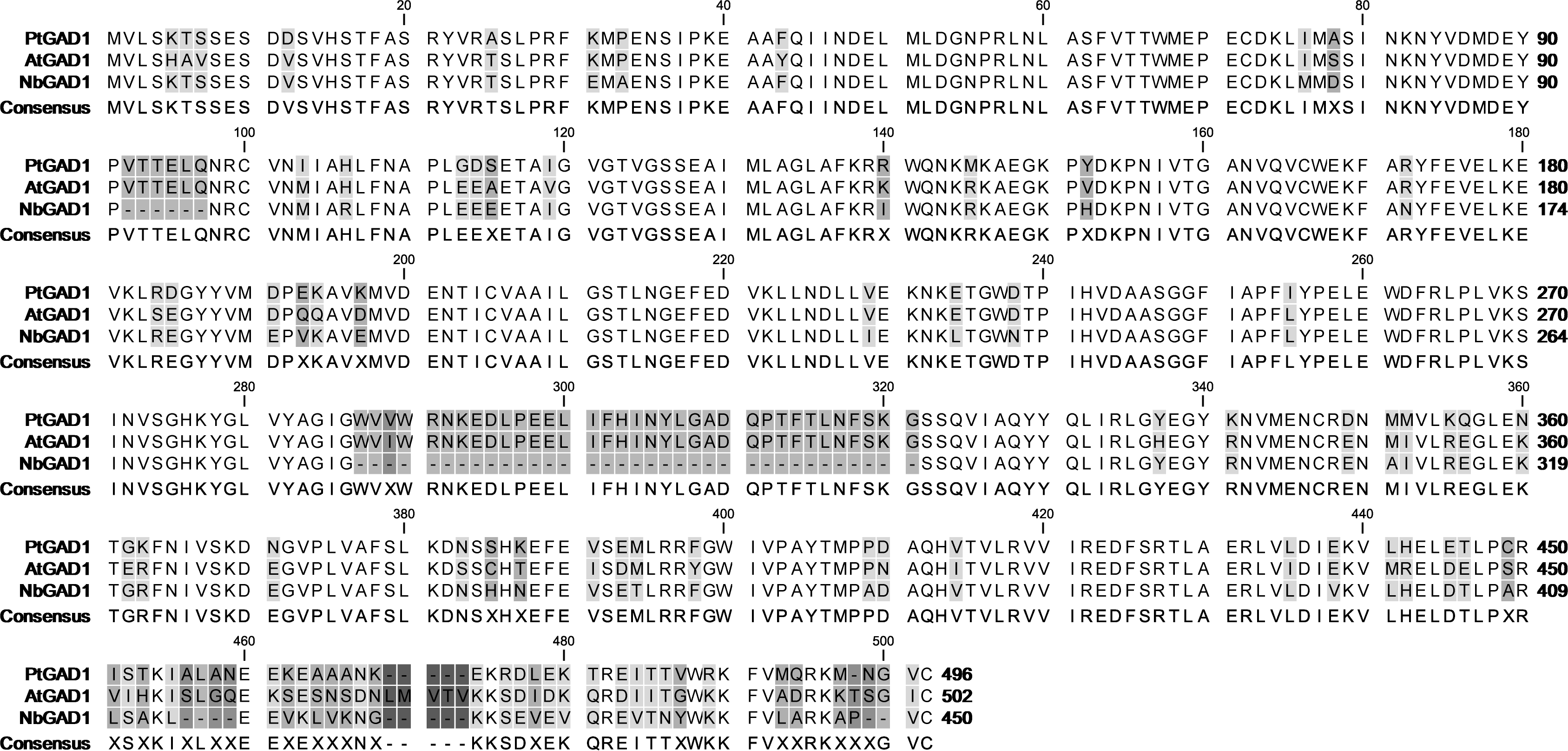

Supplement: Supplementary file 1 [file microorganisms-09-01232-s001.zip › microorganisms-1239006-supplementary/Supp Fig 2.tif]

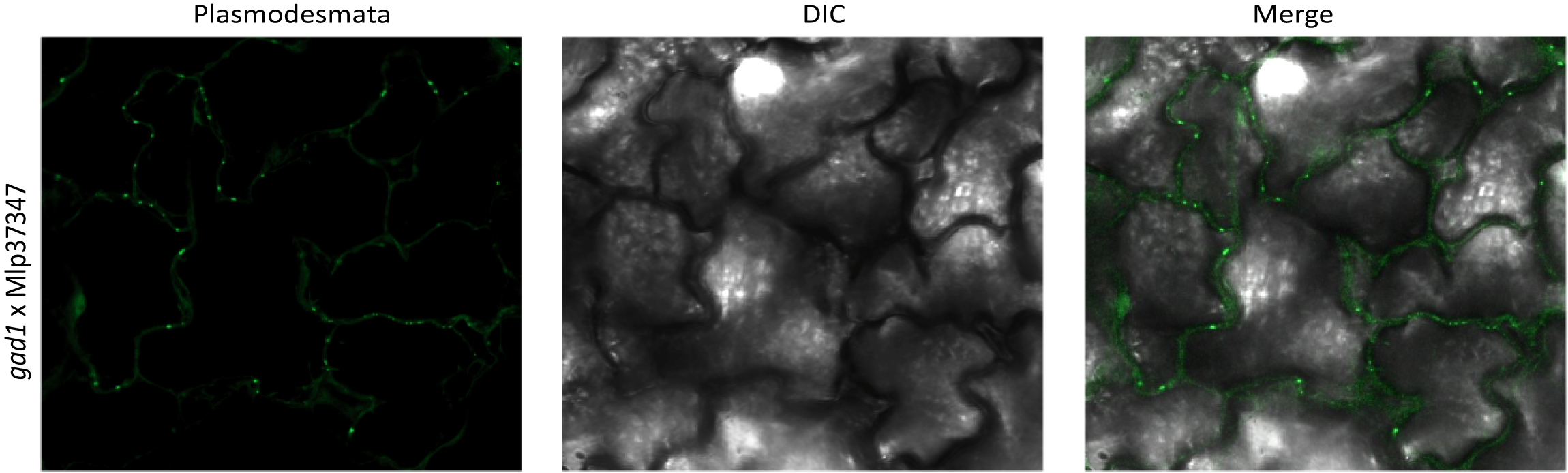

Supplement: Supplementary file 1 [file microorganisms-09-01232-s001.zip › microorganisms-1239006-supplementary/Supp Fig 3.tif]
